# 

**Published:** 1893-04

**Authors:** 


### CONTENTS.

### COMMUNICATIONS.

Treatment of a Case of Disease in the Antrum of Highmore .......... 157
The General and the Local in Dental Pathology .......... 159
Acidity of the Stomach .......... 168
A Plea for Extraction and Replantation as a means of Cure in Obstinate Alveolar Abscess .......... 169
Some Facts Relative to Diseases of the Teeth and Jaws in Inebriety .......... 175
The Removal of the Tooth Pulp by the use of Cocaine .......... 183
Collar Crowns and Preparation of Roots .......... 186
The Relationship of Food to Scorbutis in Children .......... 191

### LEGISLATION.

A Bill for an Act to Regulate the Practice of Dentistry in Wyoming .......... 195

### SELECTIONS.

Abscess of the Antrum of Highmore .......... 197
The Quack Question as Handled by the City Council of Mansfield, O .......... 198
Peroxide of Hydrogen as an Aid to Diagnosis .......... 198
Women's Medical College of Baltimore .......... 199
The American Microscopical Society .......... 200

### NOTICES.

New By-Law of the Pan-American Medical Congress .......... 201
Illinois State Dental Society and Iowa State Dental Society—Joint Meeting .......... 202
Committee on Exhibits of the World's Columbian Dental Congress .......... 202
Seventh District Dental Society of Ohio .......... 203
Kentucky State Dental Society .......... 203

### EDITORIAL.

Bibliographical—Useful Hints for the Busy Dentist .......... 204
The World's Fair—Accommodations .......... 205
The Improved Abscess Syringe .......... 206

### IN MEMORY.

Obituary .......... 207
Resolutions of the Ohio College of Dental Surgery on the Death of Dr. George Watt .......... 207
Resolutions of the Cleveland Dental Society .......... 208
Obituary .......... 208

